# Coexpression network analysis identified lncRNAs-mRNAs with potential relevance in African ancestry prostate cancer

**DOI:** 10.2144/fsoa-2021-0076

**Published:** 2021-09-22

**Authors:** Rafael Parra-Medina, Liliana López-Kleine, Sandra Ramírez-Clavijo, César Payán-Gómez

**Affiliations:** 1 Faculty of Natural Sciences, Universidad del Rosario, Bogotá, Colombia; 2 Department of Pathology, Research Institute, Fundación Univeristaria de Ciencias de la Salud, Bogotá, Colombia; 3 Deparment of Pathology, Instituto Nacional de Cancerología, Bogotá, Colombia; 4 Department of Statistics, Faculty of Science, Universidad Nacional de Colombia, Bogotá, Colombia

**Keywords:** African, ancestry, coexpression, lncRNA, mRNA, prostate cancer

## Abstract

**Aim:**

This study aims to investigate similarities and differences using lncRNA and mRNA coexpression network analysis in African ancestry (AA) and European ancestry (EA) among prostate cancer (PCa) patients.

**Methods:**

We performed weighted gene coexpression network analysis of the expression from 49 of AA and 49 of EA to identify lncRNAs-mRNAs.

**Results:**

27 lncRNAs and 36 mRNAs were highly expressed in patients of AA. Two mRNAs and their antisense lncRNAs were expressed. Additionally, seven mRNAs were DE or coexpressed and had an impact on survival.

**Conclusion:**

We present a list of lncRNAs and mRNAs that were DE and coexpressed when comparing patients of AA and EA, and these data are a resource for future studies to understand the role of lncRNAs.

## Lay abstract

This research demonstrated differences among AA and EA PCa patients. 27 lncRNAs and 36 mRNAs were highly expressed in patients of AA. 24 mRNAs were coexpressed with at least one lncRNAs. Seven mRNAs (CENPN, DNAAF3, DNASE1L2, PCDHB2, PCSK6, RTN4RL2, THBS4) were upregulated, respectively. In addition, the pathway enrichment analysis revealed two interesting signaling pathways (MAPK and Wnt) that are involved in oncogenesis and are exclusively involved in patients of AA. In summary, this study provides a resource for future investigation of the role of lncRNAs and coexpressed genes in patients of AA and EA.

Prostate cancer (PCa) is the most common tumor in men and the fifth-leading cause of cancer-related death around the world [1]. The incidence, clinical presentation and mortality of PCa vary according to race/ethnicity [2]. Globally, an increased number of PCa cases has been observed in men of African ancestry (AA) [2,3]. In the USA, African–American men are more likely to present at an earlier age, have developed advanced or metastatic PCa at diagnosis and have suboptimal outcomes after standard treatment [4,5]. Aspects related to patients, such as knowledge of the disease, low education, healthcare utilization, socioeconomic factors and genetic predisposition, have been associated with this elevated incidence [4,6].

Genetic alterations, such as mutations, changes in copy number, fusion of genes, changes in gene expression and abnormal splice variants, have been reported in PCa patients of AA [7]. In a recent study performed by Yuan *et al.* [8], through an integrative comparison of genomic and transcriptomic differences, significant differences were found between AA and European Ancestry (EA) patients. The most relevant differences were SPOP mutations (20.3% AA patients vs 10.0% EA patients), TMPRSS2-ERG fusions (29.3% AA patients vs 39.6% EA patients), PTEN deletion/losses (11.5% AA patients vs 30.2% EA patients), significant enrichment of eQTL target genes between patients of AA and EA and high expression of genes associated with immune-related pathways and PTEN/PI3K signaling in patients of AA compared with patients of EA.

The products of the human transcriptome are coding RNAs or noncoding RNAs (ncRNAs). Coding RNAs are templates used to organize the amino acids that constitute proteins during the translation process, and their structure and function are well known. Furthermore, based on their sizes, ncRNAs are classified as small ncRNAs (ncRNAs; <200 bp) and long ncRNAs (lncRNAs; >200 bp). Small ncRNAs are 20 to 30 bp in length, and based on structure/functionality, they are subdivided into *cis* regulatory (internal ribosome entry site [IRES], Leader, Riboswitch), gene (tRNA, rRNA, snRNA/snoRNA, miRNAs, among others) and intron sequences (Groups I and II) [9,10]. In medicine, miRNAs are of great interest because their expression constitutes a molecular fingerprint of different types of tumor cells, and they are possible candidates for treatment targets. miRNAs also play roles in gene expression by activating transcription [11] or inhibiting translation [12].

On the other hand, lncRNAs are classified as circular (circARN) or linear according to their structure. circRNAs are a type of single-stranded RNA that are generated by the formation of a loop in the primary pre-mRNA transcript due to back-splicing of an exon and/or intron, and the circle is closed by a covalent bond that makes it less susceptible to exonucleases. circRNAs regulate the expression of target genes during transcription and splicing as inhibitors of miRNAs, and circRNAs modulate the function of proteins by acting as decoys and encoding functional peptides [13]. In addition, linear lncRNAs are classified based on genomic localization relative to protein-encoding genes. These groups are sense, antisense, bidirectional, intronic, intergenic 3′ overlapping, macro ncRNA, process transcripts and enhancer lncRNAs [9,10]. LncRNAs are involved in different cellular processes, such as maintaining nuclear structure integrity, positively or negatively regulating gene expression by recruiting transcription factors and/or chromatin remodelling complexes to DNA targets, regulating RNA splicing, acting as decoys to sequester RNA-binding proteins (RBPs) and sequestrating miRNAs (miRNA sponges) [14,15].

Dysregulation of miRNAs and lncRNAs is widely observed in the pathogenesis of different cancer types, and these RNAs are mainly involved in tumor growth, invasion and metastasis [16–18]. LncRNAs are easily obtained for study from tissues and body fluids due to their resistance to degradation. These two types of ncRNAs are good candidate biomarkers with effects on the clinical diagnosis, prognosis and treatment of cancer. In recent years, numerous preclinical and early clinical studies have explored lncRNAs as new therapeutics [19].

In PCa, numerous miRNAs are involved in different processes that promote prostate oncogenesis, such as the cell cycle, apoptosis, epithelial–mesenchymal transition (EMT), DNA replication/repair, migration, androgen receptor suppression, metastasis and treatment resistance [20–22]. lncRNAs have also been shown to be dysregulated and associated with the pathogenesis and progression of PCa [14,18,23]. In a recent review published on the past 30 years, Ramnarine *et al.* [23] found 109 lncRNAs associated with PCa. These lncRNAs are involved in the migration and proliferation of tumoral prostate cells in different stages of disease, such as localized disease, metastatic disease or castration-resistant PCa. The molecular mechanisms targeted by these lncRNAs in PCa pathogenesis were grouped into transcriptional regulation (epigenetic modification, transcriptional activation, transcriptional inhibition, RNA decoy), post-transcriptional regulation (regulation of antisense RNA, translational regulation, mRNA stabilization, miRNA host, miRNA sponge) and posttranslational regulation (subcellular structure, protein transport, protein complex mediation). In PCa, several lncRNAs have been proposed as biomarkers because they favor both carcinogenesis and tumoral progression [24]. In the present study, a systems biology analysis was conducted to identify similarities and differences using lncRNA and mRNA coexpression network analysis.

## Materials & methods

### Data collection

The data provided by TCGA and processed for reuse by recount2 [25] were used as a primary source for data analysis. Gene counts for all prostate cancer and normal prostate tissues were downloaded. Samples annotated as having African and European ancestry by Yuan *et al.* [8] were selected. Finally, we kept all tumor samples from patients of AA, and we selected the same number of samples from patients of EA. These samples were randomly matched 1:1 (AA vs non-AA), taking into account the Gleason score obtained from the clinical information registered in The Cancer Genome Atlas Program (https://www.cancer.gov/about-nci/organization/ccg/research/structural-genomics/tcga) as a parameter.

### Data preprocessing

The transcriptomic data (mRNA and lncRNA) available in recount2 [25] were already processed with Rail-RNA as described in recount2 [25]. We performed quality control of all datasets to identify that the samples we selected had a similar distribution after and before normalization by the use of density plots and box plots. Additionally, we plotted a multidimensional plot, and we performed a visual evaluation of the localization of the samples in the first four dimensions.

### Identification of differentially expressed RNAs

The gene count data of each kind of RNA in patients of AA and EA were analyzed independently on the Galaxy web platform [26]. Data were normalized by library sizes using the trimmed mean of M-values (TMM) method. Genes without more than 1 count per million mapped reads (CPM) in at least 50% of the samples were considered not expressed and were filtered out. The limma-voom method was used to identify the differentially expressed (DE) genes. The primary factor was normal versus tumor, and the second factor was the Gleason score of the samples. An mRNA or lncRNA was selected as DE if the linear fold change was higher than the absolute value of 2 and the false discovery rate (FDR) was lower than 0.01.

### Functional enrichment analysis of mRNAs with dysregulated expression

DAVID (https://david.ncifcrf.gov/) was used to identify the functions of the selected differentially expressed genes (DEGs). The upregulated and downregulated genes in each list of DEGs were analyzed separately. The Kyoto Encyclopedia of Genes and Genomes (KEGG) was chosen for overrepresentation analysis. Pathways with p-values lower than 0.05 were selected as enriched.

### Weighted gene coexpression networks analysis

The total levels of differentially expressed mRNAs and lncRNAs in tumor samples from patients of AA compared with normal samples were added to one list. Two coexpression networks were developed using this gene list. The first list was generated for patients of AA, and the second list was generated for patients of EA.

To build the coexpression networks, we followed a typical methodology. First, the similarity matrix was calculated by identifying the Pearson correlation coefficients of the expression levels for the samples based on all possible mRNA-mRNA, lncRNA-lncRNA and lncRNA-mRNA combinations. Then, the similarity threshold was calculated with the adjacency function, which was established according to the unique characteristics of each similarity matrix [27]. The method developed by Elo was used to select the threshold [28]. This method compared the tau values for the network grouping coefficient (Co) with those expected for a random network (Cr). It uses the clustering coefficient of the real graph in comparison to a rando graph. The threshold for significant similarities is chosen so that the obtained real graph is scale-free. Finally, the adjacency matrix (2 × 2) of the network was established and allowed the representation of binary relationships. In this case, a pair of genes that exhibit coordinated gene-expression activity (coexpression) is indicated by (1); otherwise, a (0) is reported. All the weighted gene coexpression networks analysis (WGCNA) analyses were performed in an R unique environment using statistical functions (https://www.r-project.org/).

### Analysis of WGCNA

The WGCNA was plotted through network analysis using Cytoscape [29]. The identification of the differences between the AA and EA networks was calculated using the advanced network merge interface function implemented in Cytoscape.

### Survival analyses of differentially expressed genes among AA & EA & genes coexpressed with lncRNAs

Gene Expression Profiling Interactive Analysis (GEPIA;) [30] was used to calculate disease-free survival and overall survival between the DEGs coexpressed with lncRNAs. The lower and upper 50% of gene-expression levels were set as the standard for analysis. The confidence interval was 95%. High- and low-expression genes are represented in red and blue, respectively. Log-rank test results with p < 0.05 were regarded as statistically significant.

## Results

### Patient characteristics

In total, 98 PCa tissues, 49 in each group (AA and EA), and 44 normal prostate tissues (7 AA patient tissues and 37 EA patient tissues) were included. The mean age of the AA patients was 56.9 years, the mean age of the EA patients 58.8 years and the mean age of the patients from whom normal prostate tissue collected was 59.3 years. Each group included the same number of patients according to the Gleason score (GS). Nine patients had GS 3 + 3, 22 had GS 3 + 4, 11 had GS 4 + 3, 2 had GS 4 + 4, 2 had GS 4 + 5, 2 had GS 5 + 4, and 1 had GS 5 + 5. In the AA group, two patients were t2a, two were t2b, 22 were t2c, 14 were t3a, and 9 were t3b, while in the EA group, two patients were t2a, 29 were t2c, 11 were t3a, 5 were t3b, and two had no information. In the AA group, 7 were n1, 36 were n0, and 6 had no information; in the EA group, 5 were n1, 29 were n0, and 15 had no information. Only two patients died in each group. Supplementary Table 1 shows all clinical data information for the 98 PCa patients and 44 normal prostate tissues obtained from TCGA.

### Identification of differentially expressed lncRNAs & mRNAs

All the samples had adequate quality control parameters, and all of them were used for additional analysis. A total of 2419 mRNAs were differentially expressed (DE) in prostate tumor tissue samples compared with normal prostate tissue samples in PCa patients of AA. Additionally, 2296 DE mRNAs were detected in PCa patients of EA. The same analysis was performed for the gene counts of lncRNAs, and 1563 and 1304 lncRNAs were detected as DE in the tissues from patients of AA versus the normal tissues and in the tissues from patients of EA versus the normal patients, respectively. Additionally, we compared the transcriptomes of tumor samples from AA versus EA patients. The number of DE mRNAs and lncRNAs was small in the direct comparison between tumor samples from AA versus EA patients. Table 1 summarizes the number of DEGs in both comparisons.

**Table 1.** Number and direction of the change of differentially expressed mRNA and lncRNA in African ancestry and European ancestry.

	Total	Up	Down
mRNA			
TumorAA vs normal tissue	2419	702	1717
TumorEA vs normal tissue	2296	600	1696
TumorAA vs tumorEA	8	5	3
lncRNA			
TumorAA vs normal tissue	1563	708	855
TumorEA vs normal tissue	1304	593	711
TumorAA vs TumorEA tissue	22	11	11

AA: African ancestry; EA: European ancestry.

### Comparison of differentially expressed mRNAs & lncRNAs in AA & EA cancer prostate patient samples

**Figure 1.**
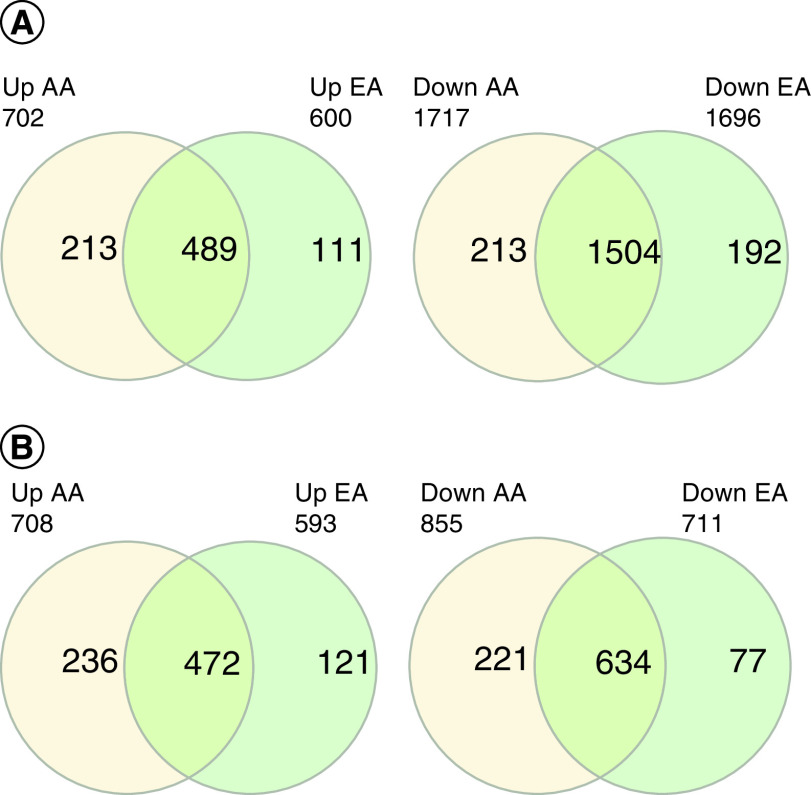
Venn diagrams show the number of (A) DE mRNAs in AA and EA. **(B)** lncRNAs in AA and EA. AA: African ancestry; EA: European Ancestry.

To obtain a general overview of the similarities and differences in the transcriptomic deregulation of AA and EA prostate cancer patient samples, we compared the lists of DE mRNAs and lncRNAs. Figure 1 shows the number of DE mRNAs and lncRNAs in both groups of patients. Common differentially expressed genes had the same direction of change.

Most DE mRNAs and lncRNAs were shared between prostate tumors from patients of AA and EA. For example, of 702 upregulated mRNAs in AA patients, 489 (69.7%) were shared with the upregulated mRNAs in EA patients. A similar result was obtained when the lists of lncRNAs were compared, following the same example: of the 708 DE lncRNAs in the samples from patients of AA, 472 (67%) of the genes were DE in common with the samples from patients of EA. Interestingly, the overlap between the lists of downregulated mRNAs and lncRNAs was even higher than that of upregulated genes. A total of 87.6% of the mRNAs and 74.2% of the lncRNAs upregulated in patients of AA were simultaneously upregulated in patients of EA.

To clarify the potential transcriptomic differences between prostate cancer samples from patients of AA and EA, we performed a direct comparison of those tumor samples. Table 2 shows the number of DE mRNAs and lncRNAs. There were only 8 mRNAs and 22 lncRNAs differentially expressed in the mentioned comparison.

**Table 2.** mRNAs and lncRNAs differentially expressed between prostate cancer samples from patients of African ancestry and European ancestry.

mRNA				lncRNA			
Gene ID	Gene symbol	logFC	Adjusted p-value	Gene ID	Gene symbol	logFC	Adjusted p-value
ENSG00000136883	KIF12	1.66	0.0024	ENSG00000274767	AC243829.1	2.08	0.0003
ENSG00000196436	NPIPB15	1.61	0.0092	ENSG00000259471	LINC01169	1.95	0.0016
ENSG00000244752	CRYBB2	1.50	0.0024	ENSG00000268181	AC073188.6	1.91	0.0038
ENSG00000182667	NTM	1.42	0.0031	ENSG00000269978	AL359881.1	1.64	0.0038
ENSG00000160973	FOXH1	1.01	0.0060	ENSG00000232283	HSD17B3-AS1	1.49	0.0088
ENSG00000104490	NCALD	-1.05	0.0083	ENSG00000275476	AC009318.4	1.43	0.0016
ENSG00000184058	TBX1	-1.28	0.0031	ENSG00000271959	AC100803.4	1.29	0.0073
ENSG00000198785	GRIN3A	-1.53	0.0083	ENSG00000262188	LINC01978	1.25	0.0062
				ENSG00000261159	AC112484.3	1.23	0.0003
				ENSG00000262877	AC110285.2	1.20	0.0046
				ENSG00000232850	PTGES2-AS1	1.20	0.0083
				ENSG00000253629	AP000426.1	-1.08	0.0029
				ENSG00000278936	AC244517.4	-1.08	0.0077
				ENSG00000277232	GTSE1-DT	-1.09	0.0016
				ENSG00000182366	FAM87A	-1.19	0.0005
				ENSG00000250604	AC098679.1	-1.19	0.0005
				ENSG00000261357	AC099518.2	-1.30	0.0069
				ENSG00000279068	AC244517.6	-1.31	0.0046
				ENSG00000249159	AC091965.1	-1.45	0.0088
				ENSG00000279472	AC244517.8	-1.76	0.0003
				ENSG00000278472	AC009268.2	-2.92	0.0080
				ENSG00000271314	AL161729.2	-3.00	0.0027

### Functional enrichment analysis of differentially expressed mRNAs

**Figure 2.**
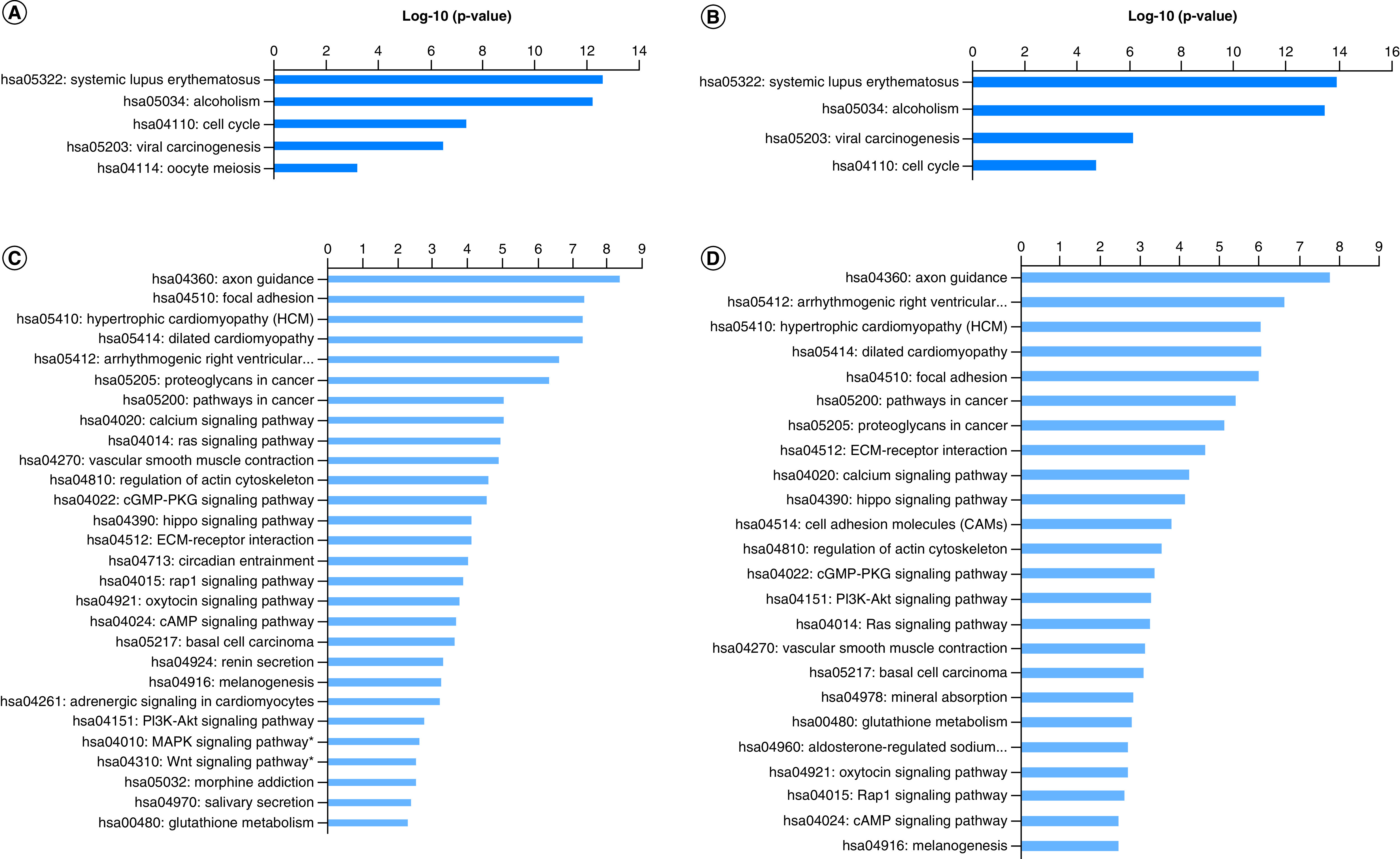
Pathway enrichment analysis for the differentially expressed between genes in patients of African ancestry and European ancestry (p < 0.05). **(A)** Enriched KEGG pathways for upregulated genes in AA. **(B)** Enriched KEGG pathways for upregulated genes in EA. **(C)** Enriched KEGG pathways for downregulated genes in AA (MAPK and WNT pathways). **(D)** Enriched KEGG pathways for upregulated genes in EA. AA: African ancestry; EA: European Ancestry; KEGG: Kyoto Encyclopedia of Genes and Genomes.

KEGG pathway enrichment analysis was then performed with the DE mRNAs observed in patients of AA and EA. Both groups presented similarities in the pathways evaluated among up- and downregulated genes. Five pathways were overrepresented among the upregulated genes of patients of AA and four pathways were overrepresented in patients of EA, and the pathway most enriched in both groups was systemic lupus erythematosus. Twenty-eight pathways were overrepresented among the downregulated genes of patients of AA and 24 in patients of EA, 21 of which were shared between patients of AA and EA. Two interesting pathways exclusively overrepresented in patients of AA were the MAPK and Wnt signaling pathways (Figure 2).

### Weight coexpression network analysis of DE mRNAs & lncRNAs in AA prostate cancer patient samples

Taking all of the previous results together, it was evident that there were few important differences in the transcriptomic deregulation of AA and EA. These differences are not sufficient to identify key molecular pathways related to the special clinical characteristics of AA prostate cancer patients.

We hypothesize that the combination of the lncRNA and mRNA expression profiles could be specific for each clinical group and that the differences could help to explain part of the differential clinical behaviour in prostate cancer patients of AA. Then, we performed a coexpression network analysis to identify critical networks of coexpressed lncRNAs-mRNAs in tumor samples from patients of AA.

First, we selected the DE mRNAs and lncRNAs in the comparison of tumor samples from patients of AA versus normal prostate samples. Second, using only the tumoral expression profiles, we calculated two coexpression networks, one for the 49 tumor samples from patients of AA and the other for the 49 tumor samples from patients of EA.

**Figure 3.**
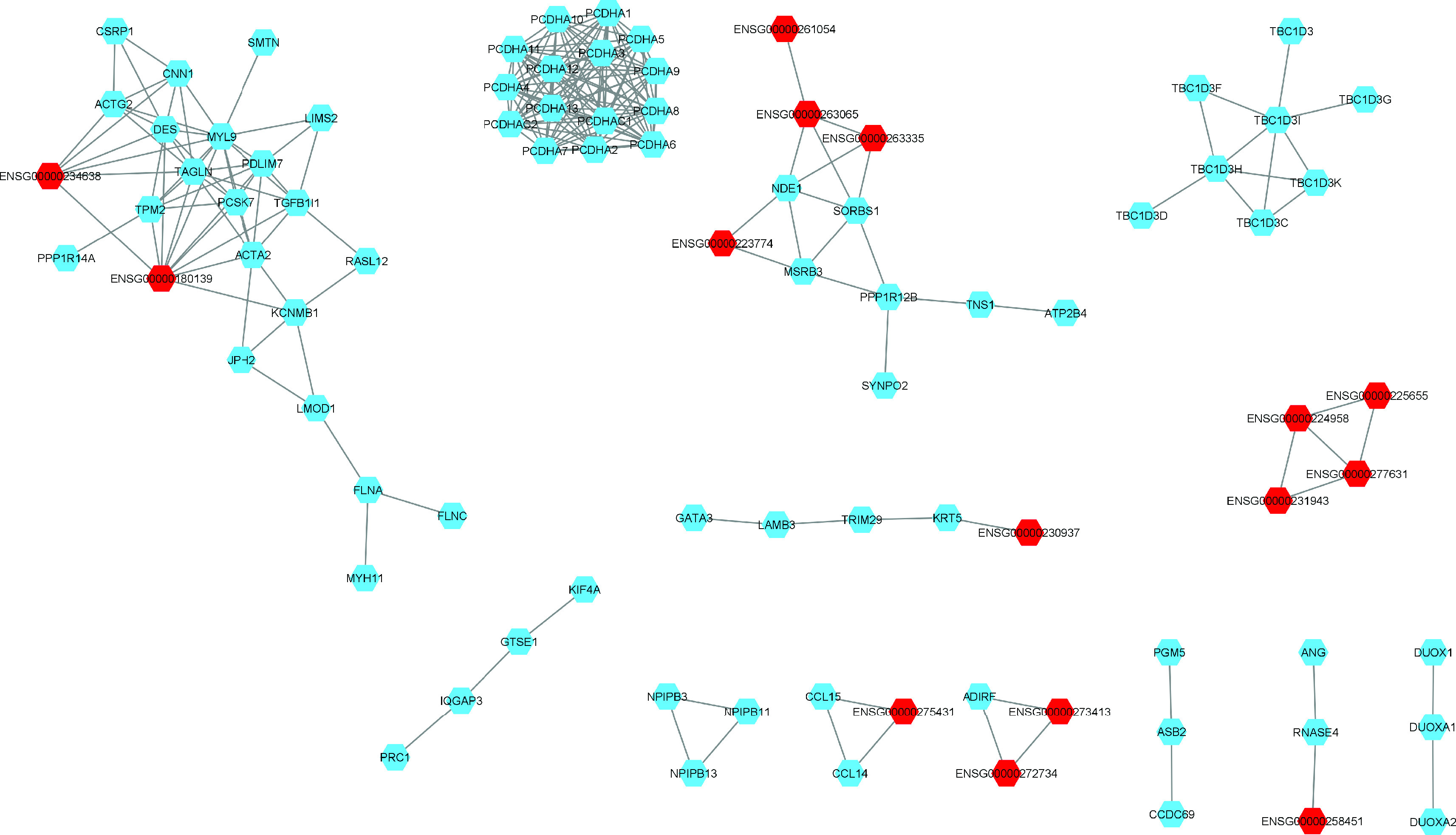
Co-expression network of DE mRNA and lncRNA in African ancestry tumor samples. lncRNA are marked in red and mRNA are marked in blue. Four networks with more than eight nodes of co-expressed genes are shown, one network co-expressed three lncRNA and another network co-expressed two lncRNA. Two tightly connected networks of the same family of genes (Protocadherin Alpha and TBC1 domain family member and 3 [TBC1D3]). In the quintet network one lncRNA was co-expressed. One quartets network was with only lncRNA co-expressed. Among the five trios networks, two had two lncRNA co-expressed and the other only one. Small networks with fewer than two nodes are not shown.

The AA network contained 224 nodes, 65 of them were lncRNA and 159 were mRNA. There were 68 pairs, six trios, two quartets, one quintet and four networks of more than eight nodes of co-expressed genes. Figure 3 shows the coexpression networks with more than five nodes as calculated with AA samples. While the EA network contained 203 nodes, 54 of them were lncRNA and 149 were mRNA. There were 52 pairs, six trios, two quartets, one quintet and three networks of more than eight nodes of co-expressed genes (Supplementary Figure 1).

**Figure 4.**
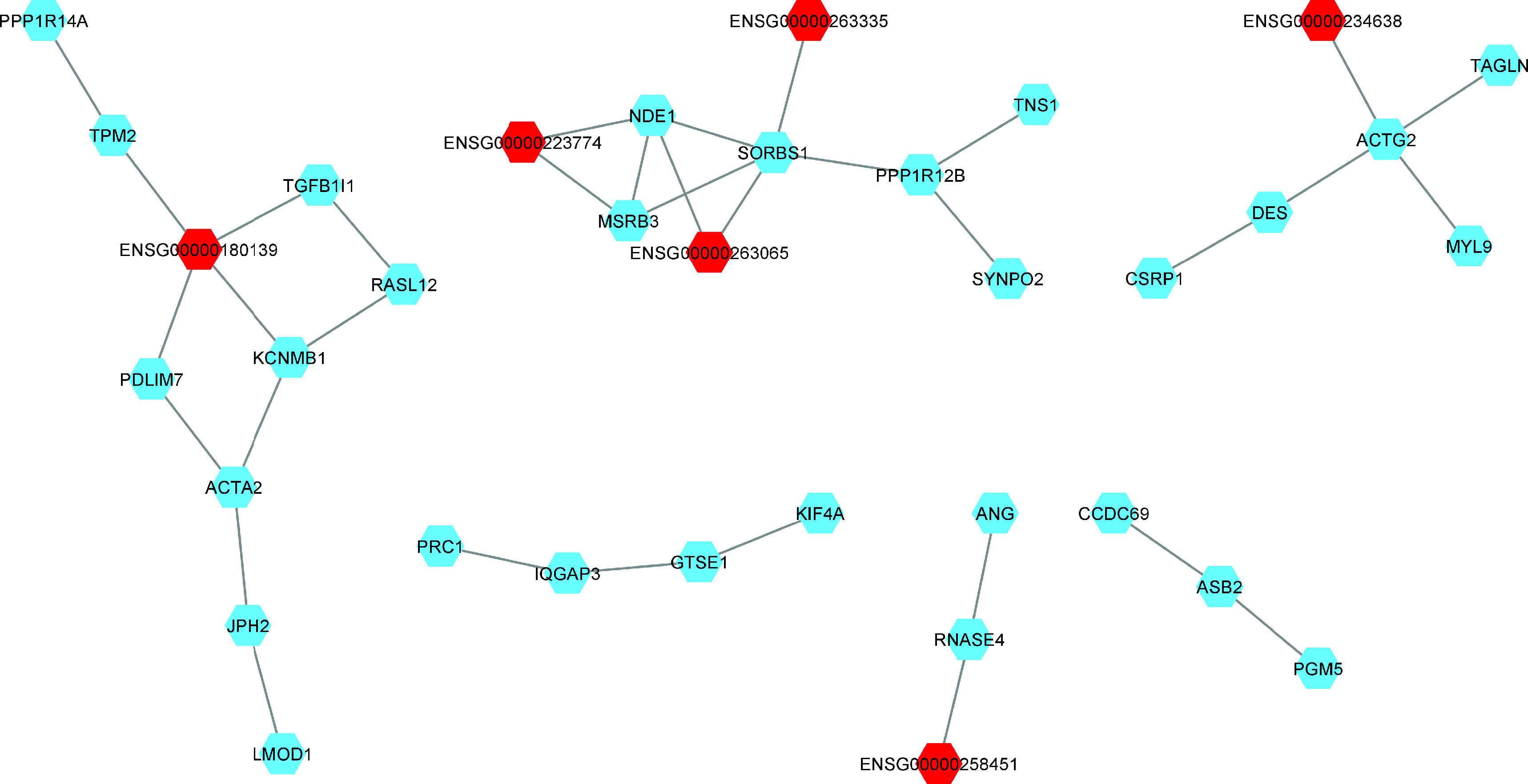
Co-expression network of the differences between African ancestry less European ancestry networks. lncRNA are marked in red and mRNA are marked in blue. One network co-expressed three lncRNA and three network co-expressed one lncRNA. Most of the small networks with fewer than two nodes are not shown.

We calculated the differences in the former network of coexpression of patients of AA with respect to the network of coexpression of patients with EA with the same mRNAs and lncRNAs but with the tumor samples from patients of EA. The results of the network difference analysis indicated the specific relations of PCa patients of AA. The instances in which patients of AA showed reduced expression compared with patients of EA included 63 nodes, 27 of which were lncRNAs and 36 of which were mRNAs. There were 29 pairs, one trio and four subnetworks with at least six nodes (Figure 4). 24 mRNAs were coexpressed with at least one lncRNAs. Seven mRNAs (CENPN, DNAAF3, DNASE1L2, PCDHB2, PCSK6, RTN4RL2, THBS4) were upregulated. THBSA4 and PCSK6 were coexpressed with their antisense lncRNAs, ENSG00000249825 and ENSG00000259172, respectively (Table 3).

**Table 3.** lncRNAs and mRNAs coexpressed in African ancestry.

Gene ID	lncRNA	logFC	Adjusted p-value		Gene ID	mRNA	logFC	Adjusted p-value
ENSG00000180139	ACTA2-AS1	-2.51	1.53E-08	Interact	ENSG00000145936	KCNMB1	-2.79	2.84E-08
ENSG00000180139	ACTA2-AS1	-2.51	1.53E-08	Interact	ENSG00000196923	PDLIM7	-1.74	3.82E-06
ENSG00000180139	ACTA2-AS1	-2.51	1.53E-08	Interact	ENSG00000140682	TGFB1I1	-2.28	4.33E-07
ENSG00000180139	ACTA2-AS1	-2.51	1.53E-08	Interact	ENSG00000198467	TPM2	-2.55	7.30E-09
ENSG00000223774	NA	-3.09	3.41E-10	Interact	ENSG00000174099	MSRB3	-2.35	6.16E-09
ENSG00000223774	NA	-3.09	3.41E-10	Interact	ENSG00000072864	NDE1	-2.94	1.19E-10
ENSG00000230729	NA	-2.34	0.000161077	Interact	ENSG00000168913	ENHO	-1.95	0.002818634
ENSG00000230937	MIR205HG	-4.12	3.32E-06	Interact	ENSG00000186081	KRT5	-3.65	1.64E-06
ENSG00000234405	NA	-2.86	3.36E-05	Interact	ENSG00000172476	RAB40A	-2.53	0.000185962
ENSG00000234477	NA	-3.42	1.81E-05	Interact	ENSG00000108244	KRT23	-3.11	0.000196113
ENSG00000234638	NA	-3.26	6.33E-10	Interact	ENSG00000163017	ACTG2	-3.19	7.14E-11
**ENSG00000249825**	**CTD-2201I18.1**	1.84	0.000143879	Interact	**ENSG00000113296**	**THBS4**	2.22	0.000308678
**ENSG00000255301**	**NA**	1.37	3.50E-05	Interact	**ENSG00000186907**	**RTN4RL2**	1.48	2.89E-06
ENSG00000258451	NA	-1.80	4.42E-08	Interact	ENSG00000258818	RNASE4	-1.55	6.29E-08
ENSG00000259018	NA	-2.68	7.19E-10	Interact	ENSG00000100842	EFS	-2.58	2.68E-08
**ENSG00000259172**	**NA**	1.27	0.000139358	Interact	**ENSG00000140479**	**PCSK6**	1.30	4.98E-05
**ENSG00000259780**	**NA**	1.97	2.91E-08	Interact	**ENSG00000167968**	**DNASE1L2**	2.00	3.32E-06
**ENSG00000260213**	**NA**	1.88	8.23E-07	Interact	**ENSG00000166451**	**CENPN**	1.78	7.19E-06
ENSG00000261707	NA	-1.29	7.90E-12	Interact	ENSG00000140876	NUDT7	-1.07	1.13E-08
ENSG00000263065	NA	-3.47	2.78E-09	Interact	ENSG00000072864	NDE1	-2.94	1.19E-10
ENSG00000263065	NA	-3.47	2.78E-09	Interact	ENSG00000095637	SORBS1	-2.52	3.83E-09
ENSG00000263335	NA	-3.38	6.01E-10	Interact	ENSG00000095637	SORBS1	-2.52	3.83E-09
ENSG00000267577	NA	1.95	5.17E-06	Interact	**ENSG00000167646**	**DNAAF3**	2.14	1.06E-06
ENSG00000267601	NA	-1.75	2.90E-07	Interact	ENSG00000178404	CEP295NL	-1.48	9.32E-05
ENSG00000271894	NA	-2.84	3.66E-10	Interact	ENSG00000055813	CCDC85A	-2.70	3.07E-07
**ENSG00000279047**	**NA**	2.15	0.00010411	Interact	**ENSG00000112852**	**PCDHB2**	2.27	7.26E-06

Genes in bold are upregulated.

### Assessment of the prognostic significance of differentially expressed genes among patients of AA & EA & genes coexpressed with lncRNAs

**Figure 5.**
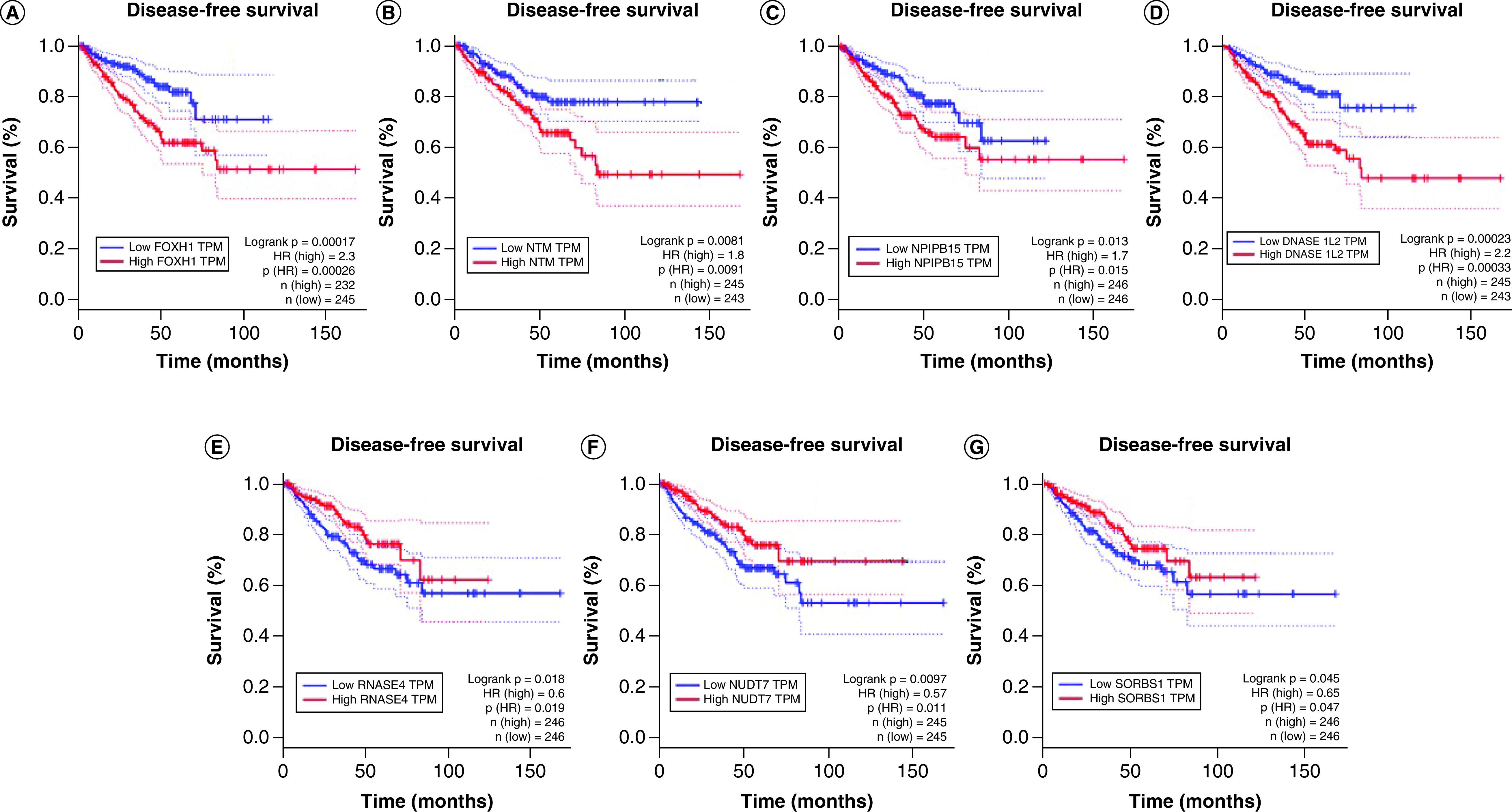
Kaplan–Meier survival plots for overall survival related to DE genes between tumors from patients of African ancestry versus tumors from patients of European ancestry and genes coexpressed with lncRNA in the African ancestry-European ancestry coexpression network. The X and Y axes represent survival time (months) and disease-free survival, respectively. The analysis was made in GEPIA.

Using GEPIA [30], which employed the PRAD dataset, the DE genes between tumors from patients of AA versus tumors from patients of EA (Table 2) and mRNA coexpressed with lncRNA in the AA-EA coexpression network (Figure 3) were identified. Among the DE genes that were associated with poor disease-free survival were FOXH1 (HR: 2.3; p: 0.0002), NTM (HR: 1.8; p: 0.009), and NPIPB15 (HR: 1.7; p: 0.015) (Figure 5 A–C). Among the genes coexpressed in the network, patients of AA, to a lesser extent than those of EA, was associated with high expression of DNASE1L2 (HR: 2.2; p: 0.0003) (Figure 5 D). and poor disease-free survival, and low expression of RNASE4 (HR: 0.6; p: 0.019), NUDT7 (HR: 0.5; p: 0.011) and SORBS1 (HR: 0.65; p: 0.047) served as protective factors for disease-free survival (Figure 5 E–G).

## Discussion

In the present study, we observed subtle differences among PCa patients of AA and EA when we compared transcriptomic dysregulation focused on individual genes. Few DE mRNAs and lncRNAs were obtained in the comparison between the two populations. In the pathway enrichment analysis, some differences between the groups were observed. Two pathways exclusive to patients of AA were the Wnt and MAPK signaling pathways. Both pathways have been associated with PCa oncogenesis due to cellular activities, including cell proliferation, differentiation, survival, death and transformation [31,32]. Interestingly, these two pathways are involved in the immune-related pathway [31,32] and regulation of the AR (androgen receptor) pathway [33,34]. Yuan *et al.* [8] observed high expression of DE mRNA-associated immune-related pathways in patients of AA and found a strong regulatory role for the AR signaling pathway in DE-lncRNAs. Variants of genes of the enzymes involved in both biosynthesis and metabolism of androgen have been observed in patients of AA [35].

Among the DE genes obtained by comparing patients of AA and EA, two upregulated genes (FOXH1 and NPIPB15) in patients of AA associated with poor disease-free survival were associated with the AR signaling pathway. FOXH1 is a member of the forkhead-box (FOX) gene family of transcription factors and participates in mediating transforming growth factor-β/activin signaling through its interaction with the Smad2-Smad4 complex [36]. FOXH1 may act as a corepressor of AR [36]. On the other hand, NPIPB15 is a member of the nuclear pore complex-interacting protein family; however, its role in PCa is unknown. The nuclear pore complex promotes the progression of PCA by increasing POM121-driven E2F1, MYC and AR nuclear import [37].

We found 1563 DE lncRNAs expressed between tumoral tissue versus normal tissue in patients of AA. In the literature, the majority of DE lncRNAs are unstudied. Yuan *et al.* [8] also observed 1868 (11.7%) DE lncRNAs, and the majority were not studied. A total of 45.9% were classified as intergenic lncRNAs, which suggests that they are transcribed from protein-coding independent transcriptional units. Additionally, a high proportion of DE lncRNAs were observed in the nucleus, which indicates that they are probably involved in gene regulation at the transcriptional level.

Due to a few differences between patients of AA and EA, we constructed two coexpression networks oriented to identify critical networks of coexpressed lncRNAs-mRNAs in tumor samples from patients of AA. The results in the first network of DE RNAs in patients of AA showed two tightly connected networks of the same family of genes, TBC1 domain family member and 3 (TBC1D3) and protocadherin alpha (PCDHA). Members of the TBC1D3 family, such as TBC1D3A or PRC7 (prostate cancer gene 17 protein), have been associated with PCa [38]. PCDHA belongs to a subset of a group of cell adhesion molecules, a cadherin superfamily. Yang *et al.* [39] observed that PCDHA is involved in the EMT axis associated with miR-193a-5p and with p53/RBM25-circAMOTL1L. These interesting networks are also present in EA networks (Supplementary Figure 1).

In the second network, where only the coexpression of lncRNAs-mRNAs in patients of AA to a lesser extent than in patients of EA was analyzed, it was observed that mRNAs and lncRNAs were still preserved. Interestingly, two of the upregulated genes (THBSA4 and PCSK6) were coexpressed with their antisense lncRNAs. THBSA4 (thrombospondin 4) was coexpressed with its antisense lncRNA (THBS4-AS1). Liu *et al.* observed high levels of THBSA4 in PCA and mainly in patients with GS >7. Cell lines (PC-3 and DU145) were observed to demonstrate reciprocal regulation of the long noncoding RNAs THBS4-003 and THBS4 to control both migration and invasion processes [40]. A recent study demonstrated that the overexpression of THBSA4 promoted self-renewal and proliferation, inhibited the apoptosis of PCa stem cells and enhanced *in vivo* tumorigenicity, which was achieved by activating the PI3K/Akt pathway [41]. THBSA4 also plays a role in the carcinogenesis of gastric cancer [42] and hepatocellular carcinoma [43]. PCSK6 is another overexpressed gene and is coexpressed with its antisense lncRNA (AC023024.1). PCSK6 is a member of the proprotein convertase family that is involved in PCa carcinogenesis [44] and was recently associated with the proliferation, invasion and migration of breast cancer cells by disturbing cell cycle arrest via the mitogen-activated protein kinase pathway [45]. A coexpression network analysis described the TGFB1I1 mRNA which is a coactivator of the AR that is associated with PCa cell differentiation, and decreased gene expression was observed to be associated with tumor progression [46].

Interestingly, given its high expression in AA and clinical impact, the DNASEL1 gene was noted. This is a member of the DNase 1-like endonuclease family and possesses keratin cell-specific endonuclease activity with an important role in DNA degradation [47]. In a recent study, it was observed that DNASEL1 is upregulated in breast carcinoma, which impacts the EMT process and has relevance in poor overall survival [48].

## Conclusion

In the present study, we observed that populations of AA and EA have many similarities in terms of DE lncRNAs and mRNAs and pathway enrichment. We provide a list of DE and coexpressed lncRNAs and mRNAs in both the AA and EA groups. The pathway enrichment analysis revealed two interesting signaling pathways (MAPK and Wnt) that are involved in oncogenesis and are exclusively involved in patients of AA. In the network analysis, lncRNAs and mRNAs were highly coexpressed in AA, and these RNAs have relevance in different pathways related to PCa oncogenesis and have been associated with survival. In summary, this study provides a resource for future investigation of the role of lncRNAs and coexpressed genes in patients of AA and EA.

**Summary points**

- We observed subtle differences among prostate cancer (PCa) patients of African ancestry (AA) and European ancestry (EA) when we compared transcriptomic dysregulation.
- The pathway enrichment analysis revealed that MAPK and Wnt are involved in oncogenesis and are exclusively involved in patients of AA.
- Two tightly connected networks of the same family of genes (TBC1 domain family member and 3 (TBC1D3) and protocadherin alpha) were expressed in AA. Members of these families have been associated with PCa.
- 24 mRNAs were coexpressed with at least one lncRNAs. Seven mRNAs (CENPN, DNAAF3, DNASE1L2, PCDHB2, PCSK6, RTN4RL2, THBS4) were upregulated. THBSA4 and PCSK6 were coexpressed with their antisense lncRNAs, ENSG00000249825 and ENSG00000259172, respectively.
- Genes differentially expressed or coexpressed as FOXH1, NTM, NPIPB15, DNASE1L2 were associated with poor disease-free survival. And low expression of RNASE4, NUDT7 and SORBS1 served as protective factors for disease-free survival.
- Further studies are required to evaluate a possible role of these mRNA-lncRNA in PCa.
