# Supplementary figures and images for: Coexpression network analysis identified lncRNAs-mRNAs with potential relevance in African ancestry prostate cancer
Source: Future Sci OA. 2021 Sep 22;7(9):FSO749. doi: 10.2144/fsoa-2021-0076 (PMC8558852; doi:10.2144/fsoa-2021-0076)

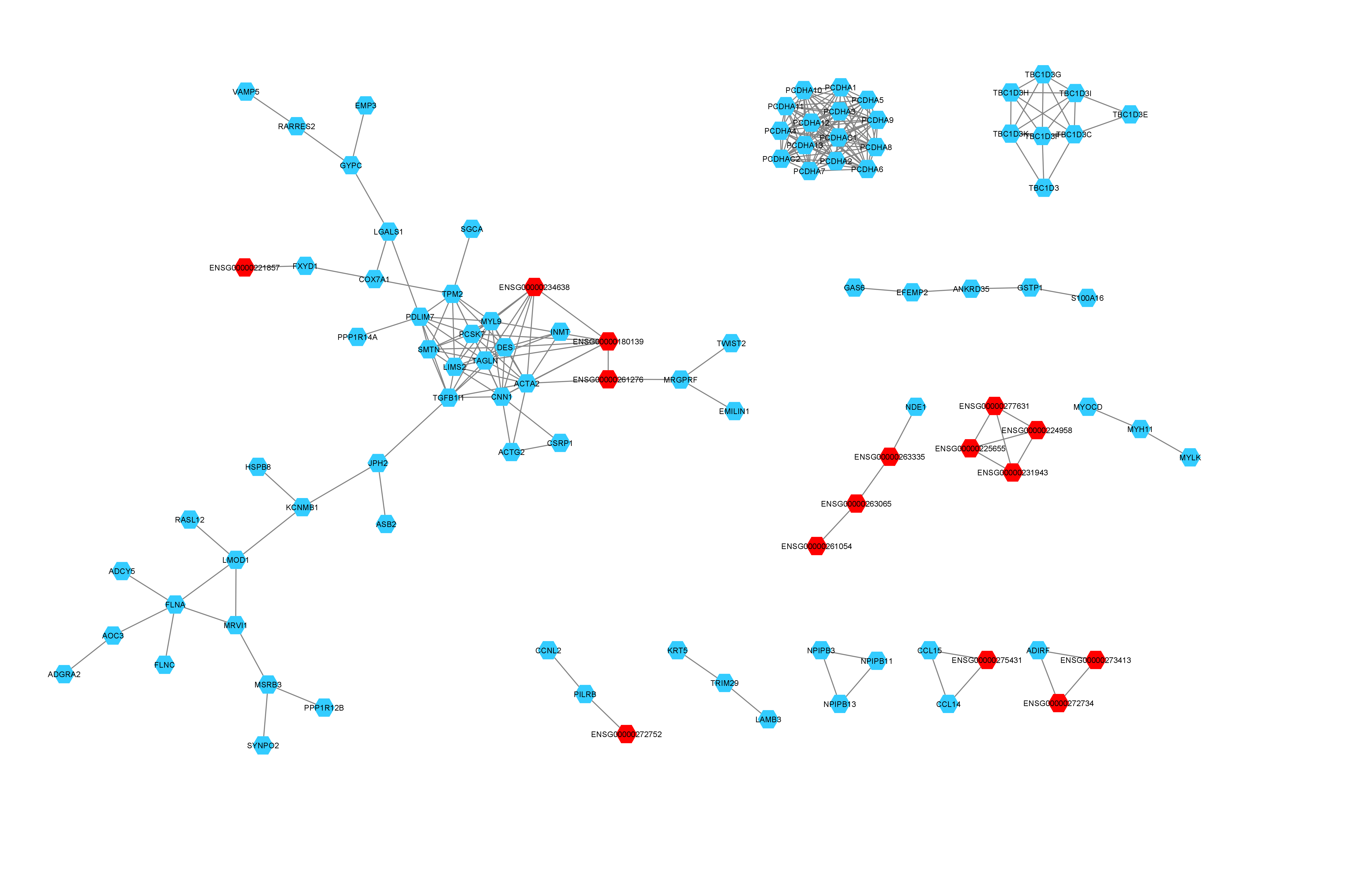

Supplement: Supplementary file 1 [file fsoa-07-749-s1.png]
